# From Severe Herpes Zoster to Rare Suid Herpesvirus Encephalitis: A New Twist of the *Varicellovirus* Genus Infection in Patients with Kidney Diseases

**DOI:** 10.7150/ijms.41952

**Published:** 2020-03-05

**Authors:** Yan Zhang, Ming Hu, Dong Wei, Hui Zhang, Bao Chu, Hao-Ming Xu, Tao Wang

**Affiliations:** 1 Department of Dermatology, the 4th Affiliated Hospital of HeBei Medical University, No.12 JianKang Road, ShiJiaZhuang 050011, P.R. China.; 2 Department of Neurology, HeBei Provincial General Hospital, No.348 West HePing Boulevard, ShiJiaZhuang 050051, P.R. China.; 3 Department of Urology, HeBei Provincial General Hospital, No.348 West HePing Boulevard, ShiJiaZhuang 050051, P.R. China.; 4 Department of Medical Imaging, HeBei Provincial General Hospital, No.348 West HePing Boulevard, ShiJiaZhuang 050051, P.R. China.; 5 Department of Respiratory Diseases, HeBei Provincial General Hospital, No.348 West HePing Boulevard, ShiJiaZhuang 050051, P.R. China.; 6 Department of Science and Education, HeBei Provincial General Hospital, No.348 West HePing Boulevard, ShiJiaZhuang 050051, P.R. China.

**Keywords:** kidney diseases, immunosuppression, opportunistic infection, herpes zoster, suid herpesvirus encephalitis

## Abstract

Both the herpes zoster virus and suid herpesvirus type 1 (SuHV-1) belong to the Varicellovirus genus of the α-herpesviridae subfamily. They may cause opportunistic infections especially in patients with kidney diseases, varying from latent illness to overt lethality. Under these circumstances, impaired renal function is both the culprit for and victim of the infection. However, fulminant eruption of severe skin herpes zoster in lupus nephritis (LN) patients under prolonged immunosuppressive therapy is rare and even more rarely seen is the SuHV-1 encephalitis in human.

Facing the evolution of these rare infections, we hence chose to review the clinical pathogenicity of these two viruses which were cognate in origin but distinct in virulence. As such, we began with the first of the two above viral diseases and proceeded with peculiar renal involvement, unique clinical symptoms and pertinent lethal risk. Of importance, LN was used to exemplify the reciprocally detrimental interactions between impaired renal function and suppressed immune response. Then in a manner similar to the gradient overlay, SuHV-1 encephalitis was discussed focusing on its neurotropic features, specific MRI findings and exclusive test of high throughput sequencing.

Our report highlighted novel presentations of the Varicellovirus genus infection by providing a productive multidisciplinary communication with pointed disclosure of the renal involvement. It may therefore be of great medical relevance and educational value for clinicians, especially the unseasoned ones, to foresee and manage similar cases in susceptible patients.

## Introduction

The increased prevalence of kidney diseases and subsequent application of new immunosuppressive agents may render a major rise of secondary opportunistic infections 1,2. Indeed, the leading cause of death in patients with systemic lupus erythematosus (SLE) was treatment-associated infection (33%), exceeding that caused by the disease itself (31%) 3. Likewise, we had in the kidney diseases patients under immunosuppressive therapy encountered tuberculosis flare-up, cryptococcal infection, Pneumocystis pneumonia and, in the hard way, lethal nocardiosis 4. Facing these grim situations, we have designed a risk score to predict pulmonary infection in patients with primary membranous nephropathy receiving the cyclosporine regimen 5. As such, we found that the profiles of these infectious complications were less distinguishable and loss of renal function an unneglectable contributory risk. Arguably, there appears to be “a new twist in old diseases”, in which viral infection deserves no less attention, if not more.

Both the herpes zoster virus and suid herpesvirus type 1 (SuHV-1) belong to the *Varicellovirus* genus of the *α-herpesviridae* subfamily 6. They mainly invade tissues originating from the ectoderm, including skin, mucosa and nervous system. The infection sites and related diseases are diverse, varying from latent illness to potential lethality 7. As the most frequent viral infection in the SLE patients, incidence of herpes zoster has been rising 8 and it was believed to reflect the evolution in SLE treatment strategies 9. In fact, treatment of the lupus nephritis (LN) depended more on the use of mycophenolate mofetil than the lower cumulative dose cyclophosphamide 10. As a result, herpes zoster had an increased tendency to disseminate and cause morbidity, especially in patients with severe SLE and/or prolonged immunosuppression. Alternatively, the SuHV-1, also known as the Aujeszky's disease virus or pseudorabies virus, possesses a more highly neurotropic feature than other herpes viruses 7. With this inherent nature, it has a tropism to infect the nervous system by transsynaptic passage and retrograde axonal transportation. Ominantly as it is, SuHV-1 encephalitis has actually “jumped” from livestock to human in sporadic cases 11 and it caused a greater lethality, due to either necrotizing lesions in the brain or aberrant host immune response.

Therefore, there is urgent need for an indispensible capability to encounter these terrible threats in patients with kidney diseases. Since impaired renal function was rather an active culprit than a mere victim 12, its reciprocally detrimental interactions with the immune suppression and infection were initially specified. Then, we chose to sequentially review severe skin herpes zoster during the long-term immunosuppressive therapy for LN and, SuHV-1 encephalitis. The rationale lies in that the two causative viruses are cognate in origin but distinct in virulence and our progressive description may give an integrated picture, covering aspects from skin infection to encephalitis. The study had acquired proper institutional ethical approval and written informed consent from the participants.

### Impaired renal function, compromised immune response and infection

**Figure 1:**
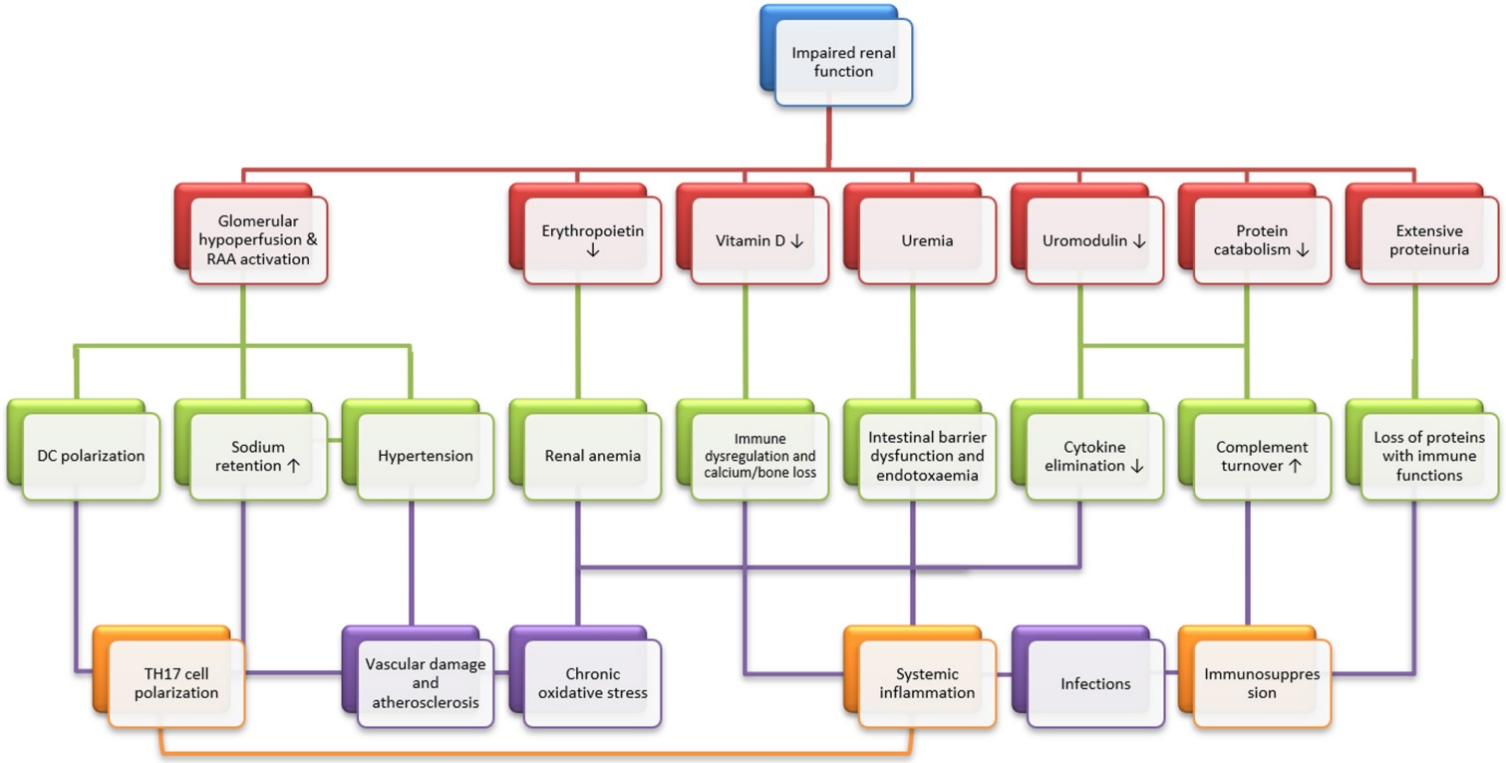
** Effects of chronic kidney diseases on systemic immunity and infection.** Chronic kidney disease (CKD) has several immediate consequences (red panels), which may result in three main immunological alterations (TH17 cell polarization, immunosuppression and systemic inflammation, orange panels) through intermediate steps (cyan panels). Briefly speaking, chronic stimulation of the renin-angiotensin-aldosterone system leads to T helper 17 (TH17) cell polarization, mainly through dendritic cell (DC) polarization. Then, uremic intestinal barrier dysfunction, vitamin D deficiency and cytokine accumulation result in systemic inflammation. Finally, systemic immunosuppression is caused by the uremic accumulation of toxic metabolic waste, increased turnover of the components of the alternative complement pathway due to impaired protein catabolism and the urinary loss of proteins with immunological functions. Also graphically depicted is the participation of key clinical consequences of CKD, which include hypertension, vascular damage and atherosclerosis, renal anemia and bone loss. These mechanisms may alone or in concert affect general immunity and infection. Modified from 2.

**Figure 2:**
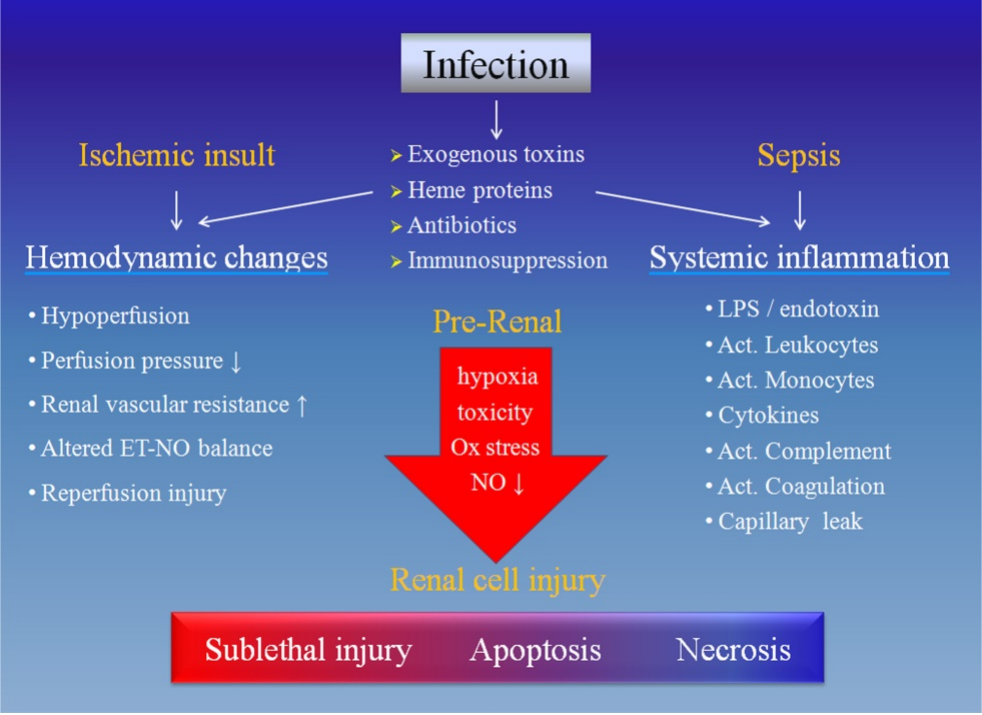
** Causative mechanisms of acute kidney injury during infection.** The infection may cause renal cell injury by hemodynamic changes due to ischemic insult and/or systemic inflammation resulted from sepsis. Each of these two components includes several 'players' and they may eventually have noxious effects on the renal cells, rendering different degrees of damage. Ox: oxidative, NO: nitric oxide, ET: endothelin, LPS: lipopolysaccharide, Act.: activated.

Their interactions may be exemplified by the SLE and LN. The SLE by its very nature is believed to be a risk factor for the herpes zoster as cellular immune response to the virus was impaired regardless of immunosuppressive regimen 13,14. Moreover, loss of renal function may exaggerate the compromised immune function and *vice versa*. As a surrogate marker for renal insufficiency, the LN may expose the patients to higher risk of herpes zoster infection 15. In this context, an elevated yet within normal range serum creatinine may play an accessory role and sensitize the effects of already existing detrimental factors on the immune function. Concurrently, the kidneys are also known 'victims' of aberrant immune function, which may confer kidney-specific damage-associated molecular patterns that cause sterile inflammation, the development of kidney-targeting autoantibodies and molecular mimicry with microbial pathogens 2. Meanwhile, declined renal function did predispose to infections (Figure 1) and the converse is also true (Figure 2). Consistently, our latest work confirmed that elevated serum creatinine was an independent risk factor for severe pulmonary infection in nephrotic syndrome patients receiving the cyclosporin regimen 5. Knowingly, the kidneys are one of the most affected organs in severe infection with 50% involvement during sepsis 16.

### Unique clinical symptoms in the immunocompromised patients

**Figure 3:**
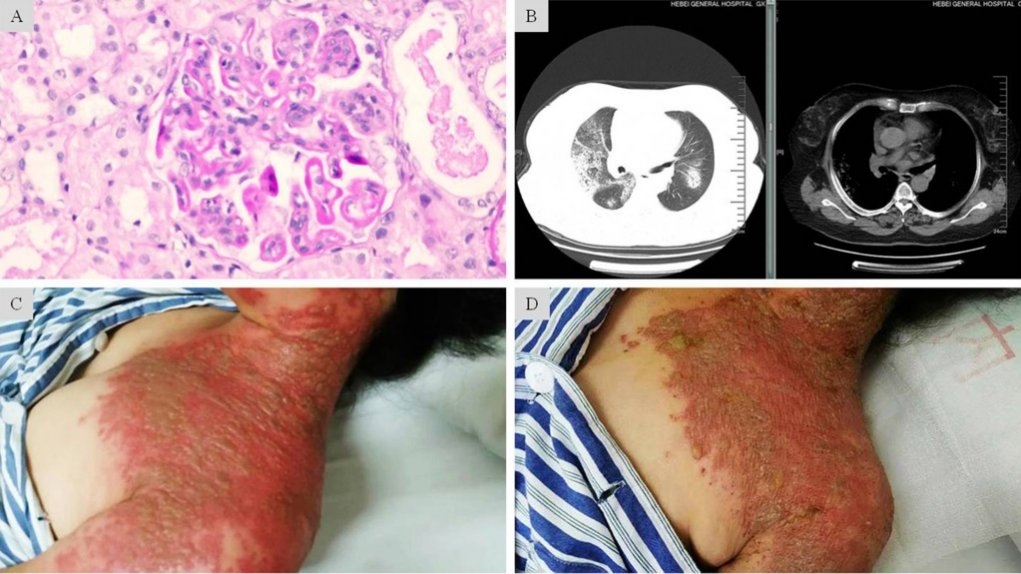
** Fulminant onset of severe herpes zoster in LN complicated with diffuse alveolar hemorrhage under prolonged immunosuppression. A:** Light microscopic findings showed glomerular endothelial and mesangial hypercellularity, deposit of the fuchsinophilic protein in subendothelial, basement membrane, subepithelial and mesangial regions. Diffuse segmental formation of wire-loops was also visible. These changes are consistent with the endocapillary proliferative lupus nephritis IV-G (A).** B:** CT scan showed in both pulmonary fields multiple scattered patchy and nodular opacity with ill-defined border. Along with the clinical features, these findings suggested diffuse alveolar hemorrhage. **C:** Grouped and fusion vesicles on an acute erythematosus base. The diffuse lesions were distributed over areas innervated by multiple sensory ganglions. **D:** Mitigated skin lesions after 7-day intravenous penciclovir and 2-day oral valaciclovir treatment. The exudation in most vesicles had “dried-up” on an ebbed erythematosus base.

The clinical symptoms of herpes zoster may be disparate between patients without and with immunosuppression 17. In the immunocompetent patients, the first symptoms of herpes zoster are usually pain and parenthesia in the involved dermatome, which often precedes the eruption by several days 18. After the preherpetic period, the rashes may slowly begin as erythematous macules, develop to vesicles within 24 hours and evolve into pustules by the third day. Of note, these lesions are generally limited to the area of skin innervated by a single sensory ganglion 19. In the immunocompromised patients, by comparison, this disease may erupt in a more violent way with markedly increased severity and cover extraordinarily large dermatome innervated by multiple sensory ganglions (Figure 3). Subsequently, incidence of complications such as visceral dissemination, postherpetic neuralgia and bacterial superinfection are also increased 20. When displaying cutaneous disseminated lesions, approximately 10% of these patients may manifest widespread and often fatal visceral dissemination 21.

### Pertinent lethal risk of viral meningitis and encephalitis

Viral meningitis caused by the herpes zoster is supposed to be the most dangerous visceral dissemination. In a study from Finland 22, 8% of the 144 immunocompetent patients diagnosed with aseptic meningitis attributed to herpes zoster and the incidence is admittedly higher in immunocompromised patients. Furthermore, it was reportedly associated with the *de novo* introduction of tocilizumab 23. As a more deadly form, there looms the SuHV-1 encephalitis or pseudorabies virus infection.

### Description of the SuHV-1 and affected systems

SuHV-1 is a double-stranded DNA herpes virus encased by capsid, tegument and envelope, as a structural mimicry of that explicitly displayed in a latest Science report 24. Of importance, glycoproteins in the envelope may compromise the immune response of the host animal by preventing the virus-infected monocytes from efficient antibody-dependent, complement-mediated lysis 25. As a result, the SuHV-1 tends to replicate rapidly with cytopathic effects to produce viral particles in a matter of hours and the said compromise was believed to be a significant determining factor in the death of infected swine 26. Humans were thought to have resistance to the SuHV-1 infections 27; however, more human infections have emerged, especially in China, in recent years 28. Yet the underlying pathological pathway for SuHV-1 in humans and the putatively inherent immune defect of the patients remain unknown. Clinically, it is mainly manifested as central nervous system (CNS) infection, followed by that of the pulmonary and hepatic systems. In addition, the virus can be transmitted to the retina through the optic nerve when it becomes reactivated 29. Reportedly, 80% and 40% of the patients in a case series of five also manifested respiratory failure requiring mechanical ventilation and lesions of the optic nerve, respectively 30.

### Focus on the SuHV-1 encephalitis

**Figure 4:**
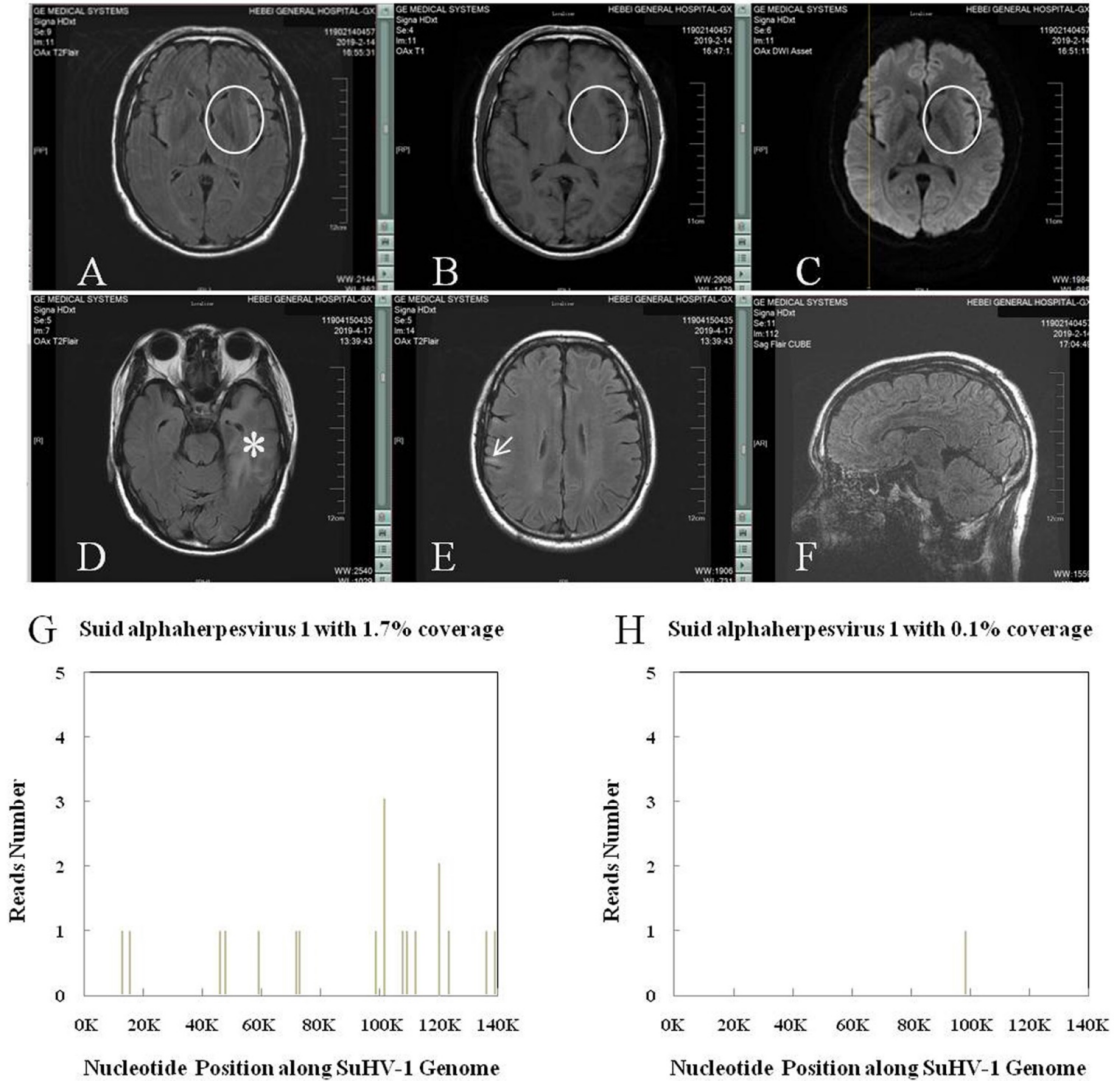
** SuHV-1 encephalitis in patient of chronic kidney disease stage 4 and the high throughput sequencing results.** A, B and C: Brain MRI showed intense T2 Flair, T1 and DWI signal changes in the left basal ganglia (circle). D and E: Brain MRI showed intense T2 Flair signal changes in the left hippocampus and temporal lobe (asterisk), and right frontoparietal lobe (arrow), respectively. F: CUBE technique found no sign of meningitis. G: Next-generation sequencing of the cerebrospinal fluid detected SuHV-1 virus with 16 unique sequence reads and 1.7% coverage. H: Next-generation sequencing of the cerebrospinal fluid detected SuHV-1 virus with 1 unique sequence reads and 0.1% coverage 22 days after treatment.

The SuHV-1 encephalitis may produce a high fever and headache, with rapid progression to signs of a CNS infection, including trembling, marked incoordination, altered mental status, seizures and, eventually, coma. Lumbar puncture should be performed early for patients with clinical signs of a CNS infection. Accordingly, the cerebrospinal fluid (CSF) generally showed elevated pressure and results similar to those of other viral encephalitis, accompanied by negative results of tryptophan test, India ink staining, Alixinlan staining and bacterial smear. Neuroimages on the brain magnetic resonance imaging (MRI) usually showed abnormal signals in the limbic system, basal ganglia and brainstem (thalamus) (Figure 4), to which the SuHV-1 had higher affinity 6. And this might explain the clinical symptoms of altered mental status and frequent seizures. By comparison, abnormal signals in encephalitis caused by herpes simplex virus-1 are usually limited to the limbic system and almost never pass claustrum to involve the basal ganglia and thalamus. Subtle difference in neuroimages can be inferred elsewhere 31. Nevertheless, next-generation sequencing of the CSF may make the definite diagnosis by identifying unique sequence reads of the SuHV-1 (Figure 4) 32. Taken together, brain MRI and next-generation sequencing of the CSF should be performed in suspected cases. Management of the SuHV-1 encephalitis should be started expeditiously that included antiviral, human immunoglobulin and symptomatic supportive treatments. In our experience, patients with visual impairment require prompt eye funduscopic examination and glucocorticoids. As a prophylactic measure, education about skin protection of persons under close contact with pigs is necessary to minimize the viral remission.

### Core tip

To learn the evolution of opportunistic *Varicellovirus* genus infection in patients with kidney diseases, our study is undoubtedly one of the most representive reports. These opportunistic infections notably shared something in common: atypical symptoms, rapid progress and sometimes fatal outcome unless attended by well-prepared medical staffs, which were exemplified in our current report. Essentially, a general picture is depicted with regard to the intimately intercalated interplay among the loss of renal function, botched immunity and the resultant infection *per se*. Based on but far beyond the scope of nephrology, our report provided a productive communication between medical disciplines and highlighted a high index of suspicion against these rare opportunistic infections in susceptible patients to ensure early diagnosis and effect timely intervention. Of great significance, the notions we narrated here are readily extended to other form of zoonostic diseases, especially those caused by new viral mutation, which may otherwise easily overrun our medical system.

## Conclusion

We hereby succinctly described the severe herpes zoster skin infection and SuHV-1 encephalitis, with special attention to the role of impaired renal function. By adding the confluent of multidisciplinary experience as a new dimension to our archive of knowledge, our work may be of enormous medical and educational value to clinicians facing the same challenges.
